# Characteristics and economic burden of frequent attenders with medically unexplained symptoms in primary care in Israel

**DOI:** 10.1080/13814788.2021.1985997

**Published:** 2021-10-11

**Authors:** Oded Hammerman, Daniel Halperin, Daniel Tsalihin, Dan Greenberg, Talma Kushnir, Yacov Ezra

**Affiliations:** a Department of Neurology, Soroka Medical Center, Be’er-Sheva, Israel; b Department of Health Policy and Management, Faculty of Health Sciences, School of Public Health, Ben-Gurion University of the Negev, Be’er-Sheva, Israel; c Soroka Clinical Research Center, Soroka Medical Center, Be’er-Sheva, Israel; d Yud Alef Clinic, Clalit Health Services, Be’er-Sheva, Israel; e Department of Psychology and Adelson School of Medicine, Ariel University, Ariel, Israel; f Faculty of Health Sciences, School of Public Health, Ben-Gurion University of the Negev, Be’er-Sheva, Israel

**Keywords:** Frequent attenders, medically unexplained symptoms, somatisation, primary healthcare, healthcare utilisation

## Abstract

**Background:**

Frequent Attenders with Medically Unexplained Symptoms (FA/MUS) are common in primary care, though challenging to identify and treat.

**Objectives:**

This study sought to compare FA/MUS to FA with organic illnesses (FA/OI) and the general clinic population (Non-FA) to understand their demographic characteristics and healthcare utilisation patterns.

**Methods:**

For this retrospective, observational study, Electronic Medical Records (EMR) were obtained from Clalit Health Services, regarding the population of a sizeable primary care clinic in Be’er-Sheva, Israel. Electronic medical records were screened to identify the top 5% of FA. FA were stratified based on whether they had OI. FA without OI were then corroborated as having MUS by their physicians. Demographics, healthcare utilisation and costs were analysed for FA/OI, FA/MUS and Non-FA.

**Results:**

Out of 594 FA, 305 (53.6%) were FA/OI and 264 (46.4%) were FA/MUS. FA/OI were older (69.1 vs. 56.4 years, *p*<.001) and costlier (ILS27693 vs. ILS9075, *p*<.001) than FA/MUS. Average costs for FA/MUS were over four times higher than Non-FA (ILS9075 vs. ILS2035, *p*<.001). The largest disparities between FA/OI and FA/MUS were in hospitalisations (ILS6998 vs. ILS2033) and surgical procedures (ILS8143 vs. ILS3175). Regarding laboratory tests, differences were smaller between groups of FA but significantly different between FA and Non-FA.

**Conclusion:**

FA/MUS are more costly than Non-FA and exhibit unique healthcare utilisation and costs patterns. FA/OI had more severe illnesses necessitating hospitalisations and surgical interventions, while FA/MUS had more investigations and tests, attempting to find an explanation for their symptoms.

KEY MESSAGES

- Close to 50% of frequent attenders (FA) have medically unexplained symptoms (FA/MUS).
- Frequent Attenders/MUS exhibit unique patterns of healthcare utilisation and costs, which can be detected using electronic medical records.
- Whereas FA with organic illness (FA/OI) had more hospitalisations, surgeries and specialist consultations, FA/MUS had higher laboratory testing costs.

## Introduction

Medically unexplained symptoms (MUS) without a clear, organic illness (OI) are caused by a complex interaction of bio-psycho-social mechanisms, comprising 33% of primary-care visits [1–4]. MUS are often transient; however, can become chronic, necessitating extensive medical attention [5]. Patients with MUS who are frequent attenders (FA) of healthcare services present a significant challenge, incurring adverse treatment outcomes, high healthcare utilisation and costs [5–6]. Several studies have assessed the economic burden of MUS. A review of the economics of MUS found excess costs ranging from $432 to $5,353 [6]. A recent US study estimated annual MUS costs at $256 billion [7].

Primary care physicians (PCP) spend much of their time with FA [8]. Some FA have OI (FA/OI), though many have MUS (FA/MUS) [8,9]. FA/MUS exhibit more psychiatric difficulties and undergo extensive, often unnecessary medical investigations, leading to inefficient utilisation of resources and potentially avoidable expenditures [10–12].

Identifying FA/MUS via electronic medical records (EMR) is advantageous, as information is easily obtainable, creating opportunities for PCP to initiate proactive care [13]. Proactive management of FA/MUS could improve care through longer consultation times and directing patients to evidence-based treatments like cognitive behaviour therapy (CBT) [12–14]. Although EMR-identification has been effective for other conditions [15,16], it is not well-established for MUS [13].

This study used EMR to characterise the demographics and unique healthcare utilisation patterns of FA/MUS and FA/OI. Characterising FA/MUS could be a first step in identifying them via EMR, creating opportunities for physicians to more effectively manage their care, decreasing healthcare utilisation and costs.

## Methods

### Study subjects

For this retrospective, observational study, EMR were obtained from Clalit Health Services (CHS), Israel’s largest health maintenance organisation (HMO). Israeli citizens receive a National List of Health Services defined by law and provided by four not-for-profit HMO. Healthcare is universal, supported by progressive taxation, supplemented by governmental funding, with voluntary premiums for supplementary insurance [17].

The study was approved by the CHS and Soroka Medical Centre institutional review boards. Data was deidentified, to maintain patient anonymity. Patients were at least 18 years of age, registered with the largest primary-care clinic in Be’er Sheva, Israel. The clinic employs 10 PCP, with 15–35 years’ experience. Interactions between PCP and the research team were minimal, focussing solely on the study.

### Study design

In accord with previous research [10], FA were defined as the top 5% of clinic attendees. Using EMR, FA were identified and stratified based on whether they had cancer, renal failure, congestive heart failure (CHF), ischaemic heart disease (IHD), cirrhosis, cerebrovascular accident (CVA), dementia, psychosis or bipolar disorder; reflecting previous diagnoses throughout primary and secondary care and differentiating between FA/OI and FA without OI. Next, to establish the existence of MUS, EMR of FA without OI were examined by their PCP, who focussed on the symptoms causing their frequent visits. Patients were considered FA/MUS only after the PCP verified their visits were due to MUS. Once FA/MUS were identified, all groups were compared (e.g. FA/MUS, FA/OI and the general clinic population – Non-FA).

### Outcomes

Utilisation and cost data were collected based on CHS’ administrative claims data. Actual CHS costs are presented. Services paid out of pocket or by supplementary insurance were not included. Utilisation rates for PCP visits are presented without costs, as PCP are salaried employees, and their costs are not captured in CHS’ administrative database. Utilisation/cost data for secondary and tertiary care were provided by CHS and reflect internal pricing estimates when services were provided directly by CHS or price rates when care was covered by CHS but provided externally. Costs are presented in Israeli Shekels (ILS); exchange rate 1 USD = 3.5 ILS. To present a more nuanced view, utilisation/cost data were analysed by type of service: (a) specialist consultations, (b) hospitalisations (visits priced by length of stay), (c) diagnostic tests (CT, MRI, etc.), (d) emergency department care (not resulting in hospitalisation), (e) laboratory tests, (f) surgeries (procedure-related group – PRG), (g) health professions’ services (physical therapy, occupational therapy, etc.), (h) medical equipment and (j) total costs (including prescribed medications). Other health conditions (e.g. diabetes, hypertension, chronic obstructive pulmonary disease and smoking) were also analysed.

### Analysis

Continuous data was presented as mean and standard deviation (SD) or median and interquartile range (IQR), as appropriate. Dichotomous data was presented as *N* and percentage. Groups were compared using Chi-square tests for categorical data and *t*-tests for continuous data.

Healthcare utilisation and cost categories were presented as mean and standard deviation (SD) or median and interquartile range (IQR). Group comparisons used *t*-test or Kruskal–Wallis test when variables were not normally distributed.

Linear regression, adjusted for sex and age, compared total costs between treatment groups. Because of their non-normal distribution, total cost data were log-transformed before the multivariable analysis. The coefficient and 95% confidence interval were back-transformed to their original scale. *p*-Values <.05 were considered statistically significant. Statistical analyses were performed using SAS 9.4 (SAS Institute Inc., Cary, NC, USA).

## Results

### Demographics

**Figure 1.**
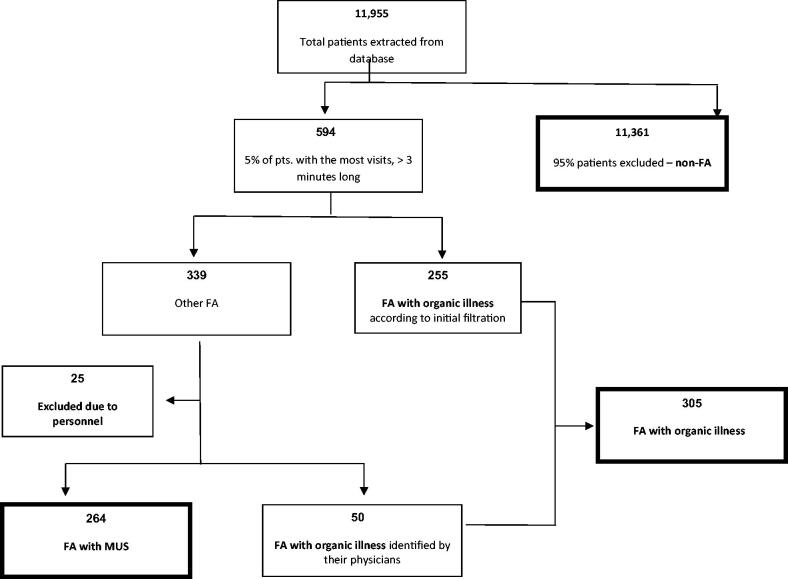
Study flow diagram.

Data from 11,955 patients (Figure 1) who received clinical care from December 2013 toDecember 2014 were analysed. The top 5% of FA included 594 patients, 255 of whom were FA/OI. PCP reviewed the remaining 339 EMR, identifying 50 additional patients with OI undetected by electronic screening and establishing FA/OI = 305 altogether. An additional 25 CHS personnel were identified and excluded from further analysis, leaving a total of FA = 569. These were deemed non-representative of FA, as their utilisation rates were due to the convenience of obtaining prescriptions and regular healthcare visits where they work. The remaining 264 files were confirmed as FA/MUS.

Almost half of FA were FA/MUS (*N* = 264, 46.4%) (Table 1). FA/MUS were similar in age to Non-FA, and younger than FA/OI (56.4 years vs. 69.1 years, *p*<.001). The most common OI were cancer (*N* = 138, 45.2%), IHD (*N* = 111, 36.4%), chronic renal failure (*N* = 93, 30.5%) and CHF (*N* = 63, 20.7%). FA/MUS presented with a number of different symptoms, such as specific pain (e.g. back, chest, neck or limb) (*N* = 160, 60.4%), symptoms of autonomic dysregulation (e.g. dizziness, hypocapnia) (*N* = 59, 22.3%), headache (*N* = 15, 5.7%), widespread pain (*N* = 14, 5.3%) and assorted symptoms (e.g. idiopathic allergies, etc.) (*N* = 10, 3.8%). Gender distribution did not differ between FA; however, there were more women in both the FA/MUS group (64.8%) and the FA/OI group (62%) than in the Non-FA group (53%; *p*<.001). FA/OI had had higher rates of other health conditions such as diabetes (46.2 vs. 26.1%; *p*<.001) and hypertension (74.8 vs. 44.7%; *p*<.001) than FA/MUS. However, FA/MUS had higher rates of smoking than FA/OI (36.4 vs. 28.9%, *p*=.05). Significantly more FA/MUS had diabetes (26.1%), hypertension (44.7%) and smoked (36.4%) than did Non-FA (9.1, 18.4, and 25.7%, *p*<.001 for all comparisons).

**Table 1.** Cohort characteristics of frequent attenders (FA) with medically unexplained symptoms (MUS), FA with organic illness (OI) and general clinic population (Non-FA).

Variable	FA/OI	FA/MUS	Non-FA	*p*-Value FA/OI vs. FA/MUS	*p*-Value Non-FA vs. FA/MUS
Number of patients (*N*)	305	264	11,361	**_**	**_**
% From amongst FA	53.6%	46.4%	**_**	**_**	**_**
Age, years					
Mean (SD)	69.1 (14.4)	56.4 (17.2)	54.2 (22.0)	<0.001	0.10
Median (IQR)	72 (61, 79)	57.5 (44, 67)	55.0 (33.0, 73.0)		
Sex (*N*, %)					
Male	116 (38%)	93 (35.2%)	5334 (47%)	0.49	<0.001
Female	189 (62%)	171 (64.8%)	6027 (53%)		
Medical background					
Diabetes	141 (46.2%)	69 (26.1%)	1027 (9.1%)	<0.001	<0.001
Hypertension	228 (74.8%)	118 (44.7%)	2073 (18.4%)	<0.001	<0.001
Chronic obstructive pulmonary disease (COPD)	21 (6.9%)	13 (4.9%)	137 (1.2%)	0.33	<0.001
Smoking	88 (28.9%)	96 (36.4%)	2098 (25.7%)	0.06	<0.001
Illness distribution for FA/OI (multiple diagnoses were possible, so numbers exceed 100%)					
Ischaemic heart disease	111 (36.4%)				
Congestive heart failure	63 (20.7%)				
Cirrhosis	4 (1.3%)				
Chronic bronchitis	7 (2.3%)				
Dementia/Alzheimer/OMS	23 (7.5%)	**_**	**_**	**_**	**_**
Psychoses	18 (5.9%)				
Schizophrenia	11 (3.6%)				
Malignancy	138 (45.2%)				
Chronic renal failure	93 (30.5%)				
Cerebrovascular accident	45 (14.8%)				

### Healthcare utilisation and costs

**Figure 2.**
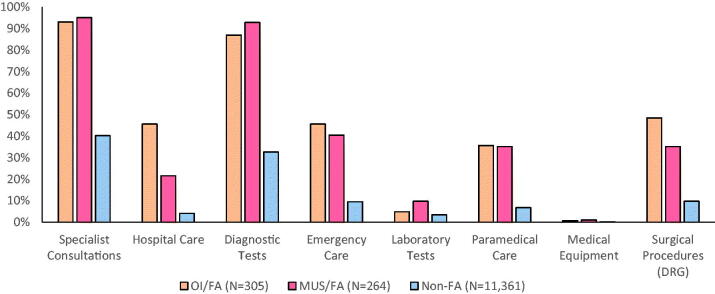
Percentage of patients utilising various healthcare services. OI: organic illness; FA: frequent attenders; MUS: medically unexplained symptoms; non-FA: non-frequent attenders (i.e. the general clinic population). This figure shows the percentage of individuals in each group using each hospital service or type of procedure.

Table 2 compares healthcare utilisation data. Both groups of FA had high average rates of PCP visits (FA/OI = 24.7 (7.6), FA/MUS = 22.3 (7.1)) when compared to Non-FA (3.9 (4.3)), as this was the inclusion criterion. In secondary care, FA/OI had significantly higher average rates of hospitalisation days (783.6 (1117.6)) and surgical procedures (836.1 (1158.2)) than either FA/MUS (299.2 (656.5), 500 (822.7); respectively) or Non-FA (55 (307.3), 128.4 (435.8); respectively), *p*<.001. However, FA/MUS had equal or higher utilisation rates when compared to FA/OI regarding specialist consultations (4234.8 (2691.7), 4554.1 (2953.2); respectively, *p*=.25), diagnostic tests (4003.8 (3035.3), 3613.1 (2814.6); respectively, *p*=.15), laboratory tests (98.5(298.5), 49.2 (216.6); respectively, *p*=.02) and health professions’ services (863.6 (1363.7), 744.3 (1205.9), respectively *p*<.001). Concurrently, when looking at the percentage of patients utilising healthcare services (Figure 2), surgeries and hospitalisations differed among FA/OI, FA/MUS and Non-FA. However, FA/OI and FA/MUS had similar patterns of specialist consultations, diagnostic and laboratory tests, health professions’ services and emergency visits.

**Table 2.** Healthcare utilisation in primary and secondary care.

Resource	Measure	FA/OI (*N* = 305)	FA/MUS (*N* = 264)	Non-FA (*N* = 11,361)	*p*-Value	
					FA/OI vs. FA/MUS	Non-FA vs. FA/MUS
Primary care						
Primary care consultations (over 12 months)	Mean (SD)	24.7 (7.6)	22.3 (7.1)	3.9 (4.6)	<.001	<.001
	Median (IQR)	23.0 (19.0, 28.0)	20.0 (18.0, 25.0)	2.0 (0.6)	.42	<.001
Secondary care						
Specialist consultations	Mean (SD) per 1,000 patients*	4554.1 (2953.2)	4234.8 (2691.7)	916.4 (1488.8)	.25	<.001
Hospital care		783.6 (1117.6)	299.2 (656.5)	55 (307.3)	<.001	<.001
Diagnostic tests		3613.1 (2814.6)	4003.8 (3035.3)	715.2 (1367.9)	.15	<.001
Emergency care		550.8 (672.4)	522.7 (713.4)	106.8 (348)	.38	<.001
Laboratory tests		49.2 (216.6)	98.5 (298.5)	37.6 (222.6)	.02	<.001
Services provided by health professionals		744.3 (1205.9)	863.6 (1363.7)	121.1 (501.5)	.66	<.001
Medical equipment		6.6 (80.8)	11.4 (106.2)	1.8 (41.9)	.54	.0005
Surgical procedures (PRG)		836.1 (1158.2)	500 (822.7)	128.4 (435.8)	<.001	<.001

*In secondary care, standard utilisation figures were generally close to 0. This is because in a given year, most patients do not utilise every type of medical service. Therefore, utilisation rates were shown for 1,000 patients to represent meaningful trends. The mean (SD) was calculated for FA/OI, FA/MUS and Non-FA and then extrapolated to 1000, for scale. Median data is not shown, as it would remain close to 0 and provide little added information.

**Figure 3.**
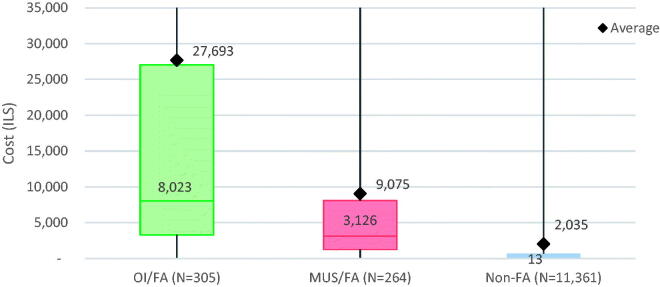
Annual total costs of study population. OI: organic illness; FA: frequent attenders; MUS: medically unexplained symptoms; non-FA: non-frequent attenders (i.e. the general clinic population). This boxplot shows both the mean and median total costs per person for each group in the study population. Both groups of FA have significantly higher mean and median costs than Non-FA. For FA/OI, there is a greater difference between mean and median costs than for FA/MUS. This is likely due to outliers with severe organic illness who have much higher annual healthcare costs. This would raise mean costs without impacting the median; a situation which is less common for patients with medically unexplained symptoms.

Utilisation rates were reflected in provider costs (Table 3, Figure 3). The total average cost for FA/MUS (9075.2 (27345.4)) was significantly less than that of FA/OI (27692.8 (50046.9)), *p*<.001 but more than four times higher than Non-FA (2035.3 (11549.9)), *p*<.001. Median (IQR) cost differences were even more striking. Median costs for FA/MUS (ILS 3,126.2 (1252.2, 8077.8)) were lower than FA/OI (ILS 8,022.8 (3307.5, 27048.4)), *p*<.001 but 200 times higher than Non-FA (ILS 13 (0, 667.9(), *p*<.001.

**Table 3.** Healthcare costs (ILS).

Variable	FA/OI (*N* = 305)		FA/MUS (*N* = 264)		Non-FA (*N* = 11,361)		*p*-Value FA/OI vs. FA/MUS*	*p*-Value Non-FA vs. FA/MUS*
	Mean (SD)	Median (IQR)	Mean (SD)	Median (IQR)	Mean (SD)	Median (IQR)		
Secondary care costs by type of service								
Specialist consultation	765.5 (668.5)	650 (273, 1048)	606.1 (497.6)	543 (232, 811.5)	118.5 (314)	0 (0, 126)	.005	<.001
Hospital care	6997.8 (15212.5)	0 (0, 6853)	2033.1 (7178.1)	0 (0, 0)	500 (6446.2)	0 (0, 0)	<.001	<.001
Diagnostic tests	1425.4 (2205.9)	685 (234, 1617)	1162.6 (1353.9)	719 (256, 1655)	180.8 (538.9)	0 (0, 86)	.70	<.001
Emergency care	458.3 (644.5)	0 (0, 619)	447.7 (791.8)	0 (0, 619)	78.3 (282.8)	0 (0, 0)	.33	<.001
Laboratory tests	3.8 (24)	0 (0, 0)	5.5 (17.2)	0 (0, 0)	3.2 (114.2)	0 (0, 0)	.02	<.001
Services provided by health professionals	174.6 (381.3)	0 (0, 153)	177.8 (356.3)	0 (0, 199.5)	22.9 (149.2)	0 (0, 0)	.89	<.001
Medical equipment	2.5 (43)	0 (0, 0)	3 (46.2)	0 (0, 0)	7.2 (743.8)	0 (0, 0)	.54	<.001
Surgical procedures (PRG)	8143.4 (23192.3)	0 (0, 1906)	3175.2 (23357.9)	0 (0, 116)	412.9 (4091.7)	0 (0, 0)	<.001	<.001
Total-cost of services (NIS)	27692.8 (50046.9)	8022.8 (3307.5, 27048.4)	9075.2 (27345.4)	3126.2 (1252.2, 8077.8)	2035.3 (11549.9)	13.0 (0, 667.9)	<.001	<.001

*Due to the abnormal distribution of these variables, *P*-values were calculated using the Kruskal-Wallis test.

Similar patterns emerged when looking at cost categories. The largest disparities between FA/OI and FA/MUS remained hospitalisations (ILS 6998 vs. ILS 2033) and surgeries (ILS 8143 vs. ILS 3175). Costs for FA/MUS were similar to FA/OI regarding specialist consultations, health professions’ services and diagnostic tests. Regarding laboratory tests, FA/MUS had higher costs than FA/OI (ILS 5.5 vs. ILS 3.8). All categories were significantly higher for FA when compared with Non-FA.

As this study sought to characterise the entire population, data from *N* = 305 (FA/OI) and *N* = 264 (FA/MUS) patients were compared to a much larger group of *N* = 11,361 (Non-FA) patients. To avoid statistical problems stemming from differences in population size, an additional analysis compared total costs of FA/MUS to a similar-sized group of Non-FA (*N* = 364) with the highest number of primary-care visits (i.e. most similar to FA). In this comparison, mean and median annual costs were still significantly higher (*p* = .0048) for the FA/MUS group (Mean (SD)=9075.2 (27345.4), median (IQR)= 3126.2 (1252.2, 8077.8)) than the similar-sized Non-FA group (Mean (SD)=8278.0 (20173.1), median (IQR)= 2138.9 (744.9, 5384.8)).

A linear regression was performed to adjust for age and gender (Table 4). The β of FA/MUS and FA/OI vs. Non-FA was 168.81 (95 CI% [98.03, 290.71], *p*<.001) and 1036.17 (95 CI% [622.60, 1724.44], *p*<.001), respectively. As a result of the regression, the β of FA/OI vs. FA/MUS was 0.01 (95 CI% [0.00, 0.01], *p*<.001) and 6.14 (95 CI% [2.94, 12.8], *p*<.001), respectively.

**Table 4.** Linear regression for group affiliation, age and gender.

Full model comparing MUS and OI to non-FA				
Variable	B	95% Confidence intervals		Pr >|t|
Intercept	42.09	33.11	53.50	<0.001
MUS vs. non-FA	168.81	98.03	290.71	<0.001
OI vs. non-FA	1036.17	622.60	1724.44	<0.001
Male vs. female	0.27	0.23	0.32	<0.001
Age (years)	0.98	0.98	0.99	<0.001

Adjusted *R*^2^=0.10.

## Discussion

### Main findings

This study was unique in looking at utilisation and cost patterns of FA from among the general population, avoiding clinical bias. Data was collected over 12 months to exclude transient symptoms rather identifying persistent FA. Almost half of FA had MUS. FA/MUS were younger, less costly and had fewer health conditions than FA/OI but were nearly five times more expensive than Non-FA. Regarding specific utilisation/cost categories, FA/OI had higher hospitalisation and surgical rates, whereas FA/MUS had higher laboratory test rates.

### Comparison with existing literature

To the best of our knowledge, no other study looked at the prevalence of FA/MUS in primary care relative to the general population, based on both EMR and PCP corroboration. Almost 50% of FA in primary care had MUS. Previous studies used diverse cut-off points and methodologies both to define FA and distinguish MUS from OI, making results difficult to compare [18,19]. Secondary care studies found similar prevalence rates, nearing 50% [20]. A UK study using a similar cut-off (top 5%) for FA, found that MUS accounted for 20% of consultations in secondary care, with higher rates in cardiology (30%), gastroenterology (50%) and neurology (50%) [21]. FA/MUS were younger and less costly than FA/OI but older and almost five times as costly as Non-FA. Similarly, Reid et al., found that FA/MUS were younger than FA/OI [10].

There were more women among FA/MUS and FA/OI. Studies found that women make up a higher percentage of patients with MUS and FA independently [2,19,22], reflecting broader gender differences in healthcare utilisation.

FA/MUS had more chronic health conditions (e.g. diabetes, hypertension) than non-FA. Similarly, Smits et al., reported that FA/MUS had high rates of chronic illness [23], especially diabetes.

Unique utilisation/cost patterns were found for FA/OI and FA/MUS. Whereas FA/OI had severe illnesses, necessitating hospitalisations and surgical interventions, FA/MUS had lower rates of hospitalisations and surgeries. Concurrently, FA/OI had higher hospital and surgical costs, as well as higher costs around specialist consultations. The only cost category in which FA/MUS had significantly higher costs was laboratory testing, perhaps reflecting patients’ continuous attempts to find explanations for their symptoms via medical investigation. This is consistent with studies showing that FA/MUS did more medical testing than FA/OI and that diagnostic procedures accounted for 40% of MUS patients’ total costs [6,10]. These patterns demonstrate that FA/MUS are a distinct population with their own utilisation profile and healthcare needs, likely due to complex interactions between patient, physician and healthcare system. Ring et al. found that during medical consultation for patients with MUS [24], physicians proposed physical interventions more often than patients did. While most patients indicated psychosocial needs,those needs were generally not picked up on. In addition to physician-patient communication, psychological factors play an important role in maintaining MUS [3]. Emotional distress and catastrophic thinking can exacerbate MUS and psychiatric illness can increase FA/MUS’ healthcare utilisation [4], as anxious patients are more likely to seek consultation [12]. This corresponds to the definition of Somatic Symptoms Disorder in the DSM-5 as ‘excessive thoughts, feelings, or behaviours related to somatic symptoms’ [25].

Ultimately, consultation behaviour of patients with FA/MUS is a complex interaction between patients’ needs and the healthcare systems’ ability to address those needs [10]. In demonstrating that FA/MUS are a distinct group with their own utilisation profile, this study strengthens the understanding that PCP could better help patients with FA/MUS by identifying which patients might benefit from focussed support, tailored to their needs, so that the most severely affected patients benefit most from additional support. Clinical guidelines for MUS recommend a stepped-care, multidisciplinary approach, wherein targeted medical investigations inform appropriate referrals to psychotherapy and additional medical professionals (e.g. physiotherapist, social worker) as necessary [26]. Early management and supportive medical treatment together with psychotherapeutic interventions (e.g. CBT, mindfulness) have been shown to improve depression, increase satisfaction and decrease addictive analgesic use for patients with MUS [27,28].

### Strengths and limitations

A strength of this study was that PCP corroborated FA/MUS. Physician evaluation has been posited as the most accurate way to identify MUS, particularly in comparison with methods like self-report surveys [29]. Ongoing physician-patient relationships have been shown to facilitate recognising MUS [30]. Physicians in this study had longstanding clinical relationships with their patients and decisions were based on thorough assessment.

Although this study looked at a large, regional centre where both the PCP and patients were representative of the local population, it was limited in that it was a single-centre study. Future studies are needed to examine whether results are generalisable across communities and cultures. Additionally, future analyses should look at how socioeconomic factors, other health conditions and behaviours influenced healthcare utilisation for patients with MUS, as these factors were not investigated. Due to the limitations of CHS’ administrative claims database, the cost of PCP visits could not be calculated. As this was the main inclusion criterion, FA/OI and FA/MUS had similar visit rates almost six-fold higher than Non-FA at the same clinic. Thus, estimates regarding FA additional healthcare costs are conservative and the difference is likely higher.

Finally, a linear regression was performed to look at the change in cost, adjusted for important risk factors, such as age and gender. Costs were found to be significantly related to group affiliation beyond the effects of age or gender. However, the R square of the model was low, meaning that not all variables influencing costs are accounted for. It is likely that patient-centred variables (e.g. specific diagnoses, emotional distress), as well as variables related to the physician-patient relationship may need to be considered to create a more robust model. As we do not yet know all of the variables predicting costs, further prospective studies will be needed.

### Implications for clinical practice

This study provides a retrospective characterisation that could be a first step in creating an EMR-based identification protocol for FA/MUS, helping PCP determine which patients might benefit from multidisciplinary care. Future studies could combine our exclusion criteria with the defining traits of FA/MUS to test potential identification algorithms. Initial criteria, such as high rates of PCP visits (>20 per year) and a negative diagnosis of OI could be used as a first stage. FA without OI could then be stratified based on age and utilisation profile to identify FA/MUS (FA/MUS were younger and had lower rates of hospitalisations, surgeries and specialist consultations, with higher rates of laboratory tests). To create an algorithm, specific cut-off points would have to be determined and their specificity and sensitivity confirmed.

## Conclusion

Almost half of FA in primary care had no organic diagnosis. These patients are a significant and costly subpopulation whose needs are not being met. FA/MUS have a unique utilisation and cost profile making it possible to identify them using EMR, helping physicians create new therapeutic alternatives to meet their needs and contain costs.
